# Supplementary material for: Polysialic acid blocks mononuclear phagocyte reactivity, inhibits complement activation, and protects from vascular damage in the retina
Source: EMBO Mol Med. 2016 Dec 22;9(2):154–66. doi: 10.15252/emmm.201606627 (PMC5286381; doi:10.15252/emmm.201606627)
Supplement: Supplementary file 1 — Appendix [file EMMM-9-154-s001.pdf]

Appendix

Polysialic acid blocks mononuclear phagocyte reactivity, inhibits complement activation and protects from vascular damage in the retina

Marcus Karlstetter[1,5], Jens Kopatz[2], Alexander Aslanidis[1], Anahita Shahraz[2], Albert Caramoy[1], Bettina Linnartz-Gerlach[2] , Yuchen Lin[3], Anika Lückoff[1], Sascha Fauser[1], Katharina Düker[2], Janine Claude[2], Yiner Wang[2], Johannes Ackermann[2], Tobias Schmidt[4], Veit Hornung[4,6], Christine Skerka[3], Thomas Langmann[1,*], and Harald Neumann[2,*]
* equal contribution

[1]Laboratory for Experimental Immunology of the Eye, Department of Ophthalmology, University of Cologne, 50931 Cologne, Germany.
[2]Institute of Reconstructive Neurobiology, University Hospital Bonn, University of Bonn, Sigmund-Freud-Str. 25, 53127 Bonn, Germany.
[3]Leibnitz Institute for Natural Product Research and Infection Biology, Beutenbergstr. 11a, 07745 Jena, Germany.
[4]Institute of Molecular Medicine, University Hospital Bonn, University of Bonn, Sigmund-Freud-Str. 25, 53127 Bonn, Germany.
[5]Therapeutic Research Group Ophthalmology, Bayer Pharma AG, Aprather Weg 18a, 42096 Wuppertal, Germany.
[6]Gene Center and Department of Biochemistry Ludwig-Maximilians-Universität München, Munich, Germany.

Table of contents

Appendix methods	-	02-05
References to Appendix methods	-	06-06
Appendix table S1	-	07-07
Appendix table S2	-	08-12
Appendix figure S1	-	13-14
Appendix figure S2	-	15-16
Appendix figure S3	-	17-17
Appendix figure S4	-	18-18
Appendix figure S5	-	19-19

Appendix methods.

Fragmentation and separation of polysialic acid. Purified α2.8-linked polysialic acid (~50-70 kDa; kindly provided by Prof. Thomas Scheper Technical Chemistry, Hannover, Germany; (Bice, Celik et al., 2013, Rode, Endres et al., 2008)) was used for fragmentation and anion exchange high-performance liquid chromatography (HPLC) separation and purification. First, samples were heated at 65°C for 90 minutes to induce spontaneous hydrolysis. Then, fragmented polySia was subjected to a 20 ml anion-exchange column (Hi Load 16/10 Q, GE-Healthcare) and separated via a HPLC system coupled to a photometric UV detector at 205/280 nm (Pharmacia Biotech), utilizing a 1 M NH_4HCO_3 buffer as solvent with a flow-rate of 1 ml/minute. The flow-through was collected in 90 tubes with a respective volume of 5 ml. Fractions were collected and tested via gel chromatography for appropriate molecular weights. PolySia fractions were loaded on a 20% polyacrylamide gel (all components Roth GmbH) and separated for 4 hours by electrophoresis at 130 V. The gel was stained via 'stains-all' solution (Sigma) over night and washed with distilled water afterwards. Low molecular weight polySia avDP20 was further analyzed by 18% Tris-Glycine polyacrylamide gel (Life Technologies GmbH; 2.5 hours electrophoresis at 125 V). Sulphated dextrans of different sizes were used as marker (TdB Consultancy). Subsequently, the gel was stained via a protocol from Goldberg and Warner for at least 2 hours at room temperature with 'stains-all' (Sigma) solution (30 mM Tris, 25% isopropanol, 7.5% formamide and 0.025% (w/v), at pH of 8.8). To clear the background, the gel was washed with distilled water including 25% isopropanol afterwards. Successful tested fractions were used for further experiments as low molecular weight polySia (here labeled as polySia avDP20). To get rid of buffer residues the samples were lyophilized and solved in PBS or distilled water. Determination of the concentration of polySia avDP20 was performed with a thiobarbituric acid-based method (Aminoff, 1961). For this purpose, polySia was pretreated with 50 mM H_2SO_4 at 80°C for 1 hour in order to hydrolyze the polymer into single n-acetylneuraminic acid (sialic acid monomers). A standard containing concentrations from 0-50 µg n-acetylneuraminic acid (Nacalai Tesque ING, Japan) was prepared. The test samples and the standard were treated with 25 µl of 25 mM periodic acid in 0.125 M H_2SO_4 and incubated at 37°C for 30 minutes. Afterwards, 20 µl of 2% sodium arsenite solution (in 0.5 N HCl) was added to each sample to reduce the excess of periodate. After 2 minutes at room temperature 200 µl of 2-thiobarbituric acid (0.1 M, pH 9) was given to the samples. Subsequently, a heating step (7.5 minutes at 99°C) was performed to induce the formation of a red colored complex. The solution was cooled on ice for 5 minutes, afterwards shaken with 500 µl/sample acid butanol (butan-1-ol plus 5% of 12 N HCl) and then centrifuged to separate the two phases. The intensity of the colorful upper phase was measured spectrophotometrically at 549 nm. Quantification was performed based on the n-acetylneuraminic acid standard.

Generation of SIGLEC11 transgenic mice by pro-nuclear injection. The SIGLEC11 splice variant of human brain microglia (Wang & Neumann, 2010) was subcloned downstream of the microglial/macrophage-specific Iba1-promoter, to generate Iba1-promoter regulated SIGLEC11 transgenic mice (Hirasawa, Ohsawa et al., 2005). The plasmid bearing the Iba1 promoter was kindly provided by Prof. Shinichi Kohsaka (Tokyo, Japan). The Iba1-promoter-SIGLEC11 vector was digested by restriction enzymes NotI and BsiEI to isolate and purify the Iba1-promoter-SIGLEC11 cassette. The target DNA fragment was isolated by 1% agarose gel electrophoresis followed by purification using the QIAquick Gel extraction kit (Qiagen) according to the manufacturer's instructions. DNA was stored at a concentration of 100 ng/µl at -80°C. Three to 5 ng/µl of DNA was used to inject into E0.5 zygotes collected from superovulated female donor mice

(F1 generation of DBA and C57BL/6J mice) and transferred to pseudopregnant recipient CD-1 mice according to established procedures. Pronuclear injection experiments were carried out by the facility of HET (University of Bonn). The SIGLEC11 transgenic mice were backcrossed for 10 generations to the C57BL/6J background before experiments were performed. Genotyping of SIGLEC11 transgenic mice was performed at the age of two to four weeks as described above. Amplified DNA was electrophoresed on a 1% agarose gel. For southern blot analysis of SIGLEC11 transgenic mice, genomic DNA was isolated from mouse tails of SIGLEC11 transgenic mice. DNA was digested by the restriction enzyme HindIII. A transgenic mouse-specific fragment (640 bp) as a probe for Southern blotting, which contains one restriction site for HindIII, was amplified by PCR using the Iba1-promoter-SIGLEC11 vector as template and the following primers: forward (5'-GAAGCTGGAGCATGGGGGAGGACTT-3'), reverse (5'-AGCAGCGTATCCACATAGCGT-3'). The DNA probe was labeled with digoxigenin and detected after fixation and hybridization using the DIG-High Prime DNA Labeling and Detection Starter Kit II (Roche) according to the manufacturer's instructions.

Animals and housing conditions. SIGLEC11-transgenic (tg) mice and wild type littermates were all on a C57Bl6/J background. Animals were maintained in an air-conditioned environment on a 12-hour light–dark schedule at 20–22°C and had free access to food and water. The health of the animals was regularly monitored and all procedures including the intravitreal injections and laser coagulation complied with the German Law on Animal Protection and the Institute for Laboratory Animal Research Guide for the Care and Use of Laboratory Animals, 1999 and the ARVO Statement for the Use of Animals in Ophthalmic and Vision Research. SIGLEC11 genotyping PCR was performed using the RED Extract-N-Amp Tissue PCR Kit (Sigma). Forward primer (5'-GGAGATGTCAGGGATGGTTC-3') and reverse primer (5'-AGCAGCGTATCCACATAGCGT-3') were used to detect the SIGLEC11 transgene. For *in vivo* procedures, mice were anesthetized by intraperitoneal (IP) injection of a mixture of Ketamine hydrochloride (100 mg/kg body weight, Ketavet, Pfizer Animal Health, New York, NY) and Xylazinhydrochlorid (5 mg/kg body weight, 2% Rompun, Bayer Health Care, Leverkusen, Germany) in 0.9% NaCl. Pupils were dilated with one drop of 0.05 % tropicamide and 0.25 % phenylephrine.

Characterization of SIGLEC11 transgenic mice. First, RT-PCR analysis of SIGLEC11 gene transcripts was performed. RNA from brain, spinal cord, liver, spleen, bone marrow and blood of the SIGLEC11 transgenic mice and littermate controls was analyzed. RNA was isolated with Trizol (Invitrogen) followed by purification with the RNeasy Mini Kit (Qiagen). Reverse transcription of RNA was performed with SuperScript III reverse transcriptase and random hexamer primers (Invitrogen). RT-PCR was carried out using the following SIGLEC11 primers: forward (5'-ACAGGACAGTCCTGGAAAACCT-3'), and reverse (5'-AGGCAGGAACAGAAAGCGAGCAG -3). Amplified DNA was electrophoresed on 1% agarose gels. For qRT-PCR the mice were injected four times daily with 1 µg/gbw LPS or vehicle control and sacrificed at day five. Brain and spleen tissue was harvested and cDNA was prepared. Quantitative transcription analysis was performed as described in the respective chapter. Second, flow cytometry analysis of SIGLEC11 was performed.
For the analysis of different tissues, organs were collected from 6-10 week-old SIGLEC11 mice and littermate controls. Preparation of the cells was done by mechanical disintegration of the respective tissue samples using cell strainers (BD Bioscience) with a size of 70 µM. Cells were washed three times in PBS to remove fat and connecting tissue before further processing. Cells

were collected and first stained with a SIGLEC-11 specific mouse monoclonal antibody (1:500, clone 3EH, described above) followed by a PE-conjugated secondary antibody. For the triple staining a Cy5 secondary antibody was used followed by direct PE- and V450-labeled CD11b (eBioscience) and CD45 (BD Horizon) antibodies, respectively. Isotype-matched control antibodies were used as negative controls. Data acquisition was performed with a FACSCalibur (BD) flow cytometer and analysis was done using FlowJo Software (BD). For immunohistochemistry analysis animals were perfused intracardially with 1× PBS followed by 4% PFA (Sigma); brain and spleen samples were collected and kept in 30% sucrose (Sigma) supplemented with 0.1% sodium azide (Sigma) until processed into frozen sections. Fixed tissue samples were sectioned (20 µm thickness) and mounted onto superfrost slides. Brain and spleen containing slides were blocked with 10% FCS and 5% normal goat serum (Invitrogen) in PBS for 1 hour at room temperature (RT). Iba1 (Dako, dil. 1:1000) and CD68 (BioRad, dil. 1:500) specific primary antibodies were incubated 2 hours at room RT. After washing with PBS, the slices were incubated for 2 hours with the corresponding secondary antibodies (Cy3-conjugated antibody, 1:200, Dianova or Alexa-488-conjugated antibody, 1:500, Invitrogen). Before mounting with Mowiol (Sigma) the samples were counterstained with DAPI (dil. 1:10000). Confocal *z*-stack images were acquired, and IBA1 and CD68-positive staining was analyzed using ImageJ software (National Institutes of Health). At least 3 images per animal were analyzed.

Siglec11/16 double knockout THP1 cell line generation. The SIGLEC11/16 double knockout THP1 cell line was generated in the laboratory of Prof. Veit Hornung using CRISPR/Cas9. An sgRNA plasmid targeting GATCAGAGACGCGCAGAGGGAGG was obtained from an arrayed genome wide library (Schmidt, Schmid-Burgk et al., 2015). The sgRNA was initially designed to target SIGLEC11, however due to the high homology of both genes, double strand breaks were introduced in both genes SIGLEC11 and SIGLEC16 resulting in frameshift mutation in all corresponding alleles. THP1 knockouts were generated as described before (Schmidt, Schmid-Burgk et al., 2016). In brief, THP1 cells were electoporated with two plasmids encoding the sgRNA and mCherry-Cas9, respectively. mCherry-positive cells were enriched by cell sorting. Sorted cells were plated under limiting dilution conditions to obtain single cell clones. The genotype was assessed by amplicon sequencing. For the analysis of the deep sequencing data the genotyping software www.OutKnocker.org was utilized (Schmid-Burgk, Schmidt et al., 2014). THP1 knockout cell clone Bc89 was selected for the experiments since it exhibited 3 deletion events in the exon 1 of SIGLEC11 and 2 deletions in the exon 1 of the SIGLEC16 gene. All other clones revealed only none to 2 genomic alterations. The removal of the SILGEC11/16 protein was confirmed via flow cytometry. Cells were differentiated as described, washed 2 times with PBS and detached mechanically. A SILGEC11-specific monoclonal antibody (clone 3EH) was used at a concentration of 2 µg/ml for 1 hour on ice. A PE fluorescence-labelled secondary antibody (Jackson Laboratories Inc, USA, 1:200) was applied for 30 minutes on ice in the dark. Control samples were incubated with an isotype control antibody. Analysis was performed with a flow cytometer (BD, FACSCalibur) and the FlowJo 8.7 Software (Tree Star Inc.).

Oligonucleotide primer sequences.
Human SIGLEC11 forward 1 (5'-CCCTGACTAAGAAGCCTGATG-3'),
Human SIGLEC11 reverse 1 (5'-GAAATGCTGATAATAAGGTCTTTGG-3')
Human SIGLEC11 forward 2 (5'- ACAGGACAGTCCTGGAAAACCT-3')
Human SIGLEC11 reverse 2 (5'- AGGCAGGAACAGAAAGCGAGCAG-3')
Human VEGFA forward (5'-GCTACTGCCATCCAATCGAG-3')

Human VEGFA reverse (5’-CTCTCCTATGTGCTGGCCTT-3’)
Human TNFSF2 (for 5’-GACAAG CCTGTAGCCCATGT-3’
Human TNFSF2 reverse (5’- AGGACCTGGGAGTAGATGAGG-3’)
Human SIGLEC11 forward 5’-CACTGGAAGCTGGAGCATGG-3’)
Human SIGLEC11 reverse (5’-ATTCATGCTGGTGACCCTGG-3’)
Human GAPDH forward (5’- CTGCACCACCAACTGCTTAG-3’)
Human GAPDH reverse (5’- TTCAGCTCAGGGATGACCTT-3’)
Mouse tnfsf2 (for 5’-TCTTCTCATTCCTGCTTGTGG-3’
Mouse tnfsf2 reverse (5’- AGGGTCTGGGCCATAGAACT-3’)
Mouse IL1beta forward (5’- CTTCCTTGTGCAAGTGTCTG-3’)
Mouse IL1beta reverse (5’- CAGGTCATTCTCATCACTGTC -3’)
Mouse Iba1 (for 5’-GAAGCGAATGCTGGAGAAAC-3’)
Mouse Iba1 reverse (5’-AAGATGGCAGATCTCTTGCC-3’)
Mouse gapdh forward (5’-ACAACTTTGGCATTGTGGAA-3’)
Mouse gapdh reverse (5’-GATGCAGGGATGATGTTCTG-3’)

References for the Appendix methods.

Aminoff D (1961) Methods for the quantitative estimation of N-acetylneuraminic acid and their application to hydrolysates of sialomucoids. Biochem J 81: 384-92

Bice I, Celik H, Wolff C, Beutel S, Zahid M, Hitzmann B, Rinas U, Kasper C, Gerardy-Schahn R, Scheper T (2013) Downstream processing of high chain length polysialic acid using membrane adsorbers and clay minerals for application in tissue engineering. Engineering in Life Sciences 13: 140-148

Rode B, Endres C, Ran C, Stahl F, Beutel S, Kasper C, Galuska S, Geyer R, Muhlenhoff M, Gerardy-Schahn R, Scheper T (2008) Large-scale production and homogenous purification of long chain polysialic acids from E. coli K1. J Biotechnol 135: 202-9

Hirasawa T, Ohsawa K, Imai Y, Ondo Y, Akazawa C, Uchino S, Kohsaka S (2005) Visualization of microglia in living tissues using Iba1-EGFP transgenic mice. J Neurosci Res 81: 357-62

Schmid-Burgk JL, Schmidt T, Gaidt MM, Pelka K, Latz E, Ebert TS, Hornung V (2014) OutKnocker: a web tool for rapid and simple genotyping of designer nuclease edited cell lines. Genome Res 24: 1719-23

Schmidt T, Schmid-Burgk JL, Ebert TS, Gaidt MM, Hornung V (2016) Designer Nuclease-Mediated Generation of Knockout THP1 Cells. Methods in molecular biology 1338: 261-72

Schmidt T, Schmid-Burgk JL, Hornung V (2015) Synthesis of an arrayed sgRNA library targeting the human genome. Sci Rep 5: 14987

Wang Y, Neumann H (2010) Alleviation of neurotoxicity by microglial human Siglec-11. J Neurosci 30: 3482-8

Appendix Table S1.

Flow cytometry analysis of SIGLEC11 expression (percentage of positive cells) of different organs from wild type and humanized SIGLEC11 transgenic mice. Data are presented as mean +/-SEM.

Organ	SIGLEC11 transgenic mice	Wild type mice
Brain	11.46 +/- 1.65 %	2.22 +/- 0.8 %
Liver	19.23 +/- 3.99 %	1.84 +/- 0.23 %
Retina	11.75 +/- 1.63 %	2.48 +/- 0.28 %
Spinal Cord	9.83 +/- 2.21 %	2.98 +/- 0.92 %
Bone Marrow	1.02 +/- 0.59 %	0.87 +/- 0.5 %
Blood	1.29 +/- 0.73 %	1.20 +/- 0.18 %

Appendix Table S2.

Summary of the statistical analysis for the figures.

Figure	group		n-number /group	compared groups	p-value	*
**Fig1B**	A	WT PBS	24	A - C	p = 0.0147	*
**RPE**	B	WT polySia 0.2 µg	37	B - E	p = 0.0046	**
	C	WT polySia 3 µg	20	D - E	p = 0.0071	**
	D	TG PBS	25	D - F	p < 0.0001	***
	E	TG polySia 0.2 µg	35			
	F	TG polySia 3 µg	31			
**Fig1D**	A	WT PBS	6	A - C	p = 0.0052	**
**Microglia**	B	WT polySia 0.2 µg	8	B - E	p = 0.0005	***
	C	WT polySia 3 µg	6	D - E	p = 0.0159	*
	D	TG PBS	8	D - F	p = 0.001	**
	E	TG polySia 0.2 µg	7			
	F	TG polySia 3 µg	6			

Figure	group		n-number /group	compared groups	p-value	*
**Fig2B**	A	WT PBS	20	A - C	p = 0.0193	*
**FFA**	B	WT polySia 0.2 µg	17	B - E	p < 0.0001	***
	C	WT polySia 3 µg	9	D - E	p < 0.0001	***
	D	TG PBS	16	D - F	p = 0.0002	***
	E	TG polySia 0.2 µg	20			
	F	TG polySia 3 µg	15			
**Fig2D**	A	WT PBS	12	A - B	p < 0.0001	***
**MAC**	B	WT polySia 0.2 µg	12	A - C	p < 0.0001	***
	C	WT polySia 3 µg	7	A - E	p < 0.0001	***
	D	TG PBS	6	A - F	p < 0.0001	***
	E	TG polySia 0.2 µg	10	D - E	p = 0.0004	***
	F	TG polySia 3 µg	12	D - F	p < 0.0001	***

Figure	group		n-number /group	compared groups	p-value	*
**Fig3A**	A	WT No treatment	7	C - A	p < 0.0001	***

**qRT TNFSF2**	B	WT PolySia avDP20	4	C - B	p = 0.0002	***
**THP1**	C	WT LPS	7			
	D	WT LPS/PolySia avDP20 0.15µM	3	C - D	p = 0.009	**
	E	WT LPS/PolySia avDP20 1.5µM	5	C - E	p = 0.002	**
	A	KO No treatment	5	C - A	p = 0.01	**
	B	KO PolySia avDP20	4	C - B	p = 0.122	
	C	KO LPS	7			
	D	KO LPS/PolySia avDP20 0.15µM	3	C - D	p = 1.000	
	E	KO LPS/PolySia avDP20 1.5µM	4	C - E	p = 1.000	
**Fig3B**	A	WT No treatment	8	C - A	p < 0.0001	***
**ELISA TNFSF2**	B	WT PolySia avDP20	5	C - B	p < 0.0001	***
**THP1**	C	WT LPS	7			
	D	WT LPS/PolySia avDP20 0.15µM	5	C - D	p < 0.002	**
	E	WT LPS/PolySia avDP20 1.5µM	4	C - E	p = 0.0003	***
	A	KO No treatment	6	C - A	p < 0.0001	***
	B	KO PolySia avDP20	5	C - B	p < 0.0001	***
	C	KO LPS	7			
	D	KO LPS/PolySia avDP20 0.15µM	5	C - D	p = 1.000	
	E	KO LPS/PolySia avDP20 1.5µM	5	C - E	p = 1.000	
**Fig3C**	A	WT No treatment	6	C - A	p < 0.0001	***
**qRT VEGF**	B	WT PolySia avDP20	5	C - B	p < 0.0001	***
**THP1**	C	WT LPS	5			
	D	WT LPS/PolySia avDP20 0.15µM	5	C - D	p = 0.0002	***
	E	WT LPS/PolySia avDP20 1.5µM	5	C - E	p < 0.0001	***
	A	KO No treatment	6	C - A	p = 0.022	*
	B	KO PolySia avDP20	5	C - B	p = 0.063	
	C	KO LPS	5			
	D	KO LPS/PolySia avDP20 0.15µM	5	C - D	p = 1.000	
	E	KO LPS/PolySia avDP20 1.5µM	5	C - E	p = 1.000	
**Fig3D**	A	WT No treatment	9	C - A	p < 0.0001	***
**ELISA VEGF**	B	WT PolySia avDP20	6	C - B	p = 0.009	**
**THP1**	C	WT LPS	9			
	D	WT LPS/PolySia avDP20 0.15µM	5	C - D	p = 0.043	*

	E	WT LPS/PolySia avDP20 1.5µM	7	C - E	p < 0.0001	***
	A	KO No treatment	9	C - A	p = 0.349	
	B	KO PolySia avDP20	6	C - B	p = 0.249	
	C	KO LPS	7			
	D	KO LPS/PolySia avDP20 0.15µM	6	C - D	p = 1.000	
	E	KO LPS/PolySia avDP20 1.5µM	7	C - E	p = 1.000	
**Fig3E**	A	WT debris	6	A-C	p = 0.028	*
	B	WT polySia 0.15	6			
	C	WT polySia 1.5	6	C-F	p = 0.000121	***
	D	KO debris	6			
	E	KO polySia 0.15	6			
	F	KO polySia 1.5	6			
**Fig3F**	A	WT untreated	6	A-B	p = 0.037	*
	B	WT debris	6	B-D	p = 0.001	***
	C	WT polySia 0.15	6			
	D	WT polySia 1.5	6	D-H	p = 0.001	***
	E	KO untreated	6			
	F	KO debris	6			
	G	KO polySia 0.15	6			
	H	KO polySia 1.5	6			

**Figure**	**group**		**n-number /group**	**compared groups**	**p-value**	*****
**Fig3G**	A	Buffer	3			
**C3b**	B	Control	3			
	C	HI-NHS	3			
	D	NHS	3			
	E	NHS+0.15µM polySia	3	D - E	p=0.7707	ns
	F	NHS+0.5µM polySia	3	D - F	p=0.9631	ns
	G	NHS+1.5µM polySia	3	D - G	p=0.8308	ns
	H	NHS+5µM polySia	3	D - H	p=0.5755	ns
	I	NHS+15µM polySia	3	D - I	p=0.5700	ns
	J	NHS+50µM polySia	3	D - J	p=0.1037	ns
**Fig3H**	A	Buffer	4			
**C3b**	B	Control	4			
	C	HI-NHS	4			
	D	NHS	4			
	E	NHS+0.15µM polySia	4	D - E	p=0.0026	**
	F	NHS+0.5µM polySia	4	D - F	p=0.0036	**

	G	NHS+1.5µM polySia	4	D - G	p=0.0004	***
	H	NHS+5µM polySia	4	D - H	p=0.0048	**
	I	NHS+15µM polySia	4	D - I	p=0.003	**
	J	NHS+50µM polySia	4	D - J	p=0.0014	**

Figure	group		n-number /group	compared groups	p-value	*
**FigEV3A**	A	WT No treatment	7	C - A	p < 0.0001	***
**qRT TNFSF2**	B	WT PolySia avDP20	5	C - B	p < 0.0001	***
**Microglia**	C	WT LPS	7			
	D	WT LPS/PolySia avDP20 0.15µM	4	C - D	p = 1.000	
	E	WT LPS/PolySia avDP20 1.5µM	4	C - E	p = 0.001	***
	A	KO No treatment	5	C - A	p < 0.0001	***
	B	KO PolySia avDP20	5	C - B	p < 0.0001	***
	C	KO LPS	5			
	D	KO LPS/PolySia avDP20 0.15µM	4	C - D	p = 1.000	
	E	KO LPS/PolySia avDP20 1.5µM	5	C - E	p = 1.000	
**FigEV3D**	A	WT debris	4	A-C	p = 0.044	*
	B	WT polySia 0.15	4			
	C	WT polySia 1.5	4	C-F	p = 0.009	**
	D	KD debris	4			
	E	KD polySia 0.15	4			
	F	KD polySia 1.5	4			
**FigEV3E**	A	WT untreated	5	A-B	p = 0.026	*
	B	WT debris	5	B-D	p = 0.004	**
	C	WT polySia 0.15	5			
	D	WT polySia 1.5	5	D-H	p = 0.031	*
	E	KD untreated	5			
	F	KD debris	5			
	G	KD polySia 0.15	5			
	H	KD polySia 1.5	5			
**FigEV3F**	A	untreated	5			
	B	debris	5	B-D	p = 0.018	*
	C	polySia 0.15	5			
	D	polySia 1.5	5			
	E	untreated +SOD1	5			
	F	debris + SOD1	5	B-F	p = 0.024	*
	G	polySia 0.15 + SOD1	5			
	H	polySia 1.5 + SOD1	5			

	I	untreated + trolox	5			
	J	debris + trolox	5			
	K	polySia 0.15 + trolox	5			
	L	polySia 1.5 + trolox	5			

**Figure**	**group**		**n-number /group**	**compared groups**	**p-value**	*****
**FigEV4B**	A	Control	3	E - A	p < 0.0001	***
	B	PolySia avDP20	8	E - B	p < 0.0001	***
	C	Control/HI-NHS	8	E - C	p < 0.0001	***
	D	PolySia avDP20/HI-NHS	6	E - D	p < 0.0001	***
	E	Control/NHS	3			
	F	PolySia avDP20/NHS	4	E - F	p = 0.00019	***
**FigEV4C**	A	NHS + PI	3			***
	B	NHS + PSA(0,05uM)	3	A - B	p = 1.000	
	C	NHS + PSA(0,15uM)	3	A - C	p = 1.000	
	D	NHS + PSA(0,5uM)	3	A - D	p = 1.000	
	E	NHS + PSA(1,5uM)	3	A - E	p < 0.0001	***
	F	NHS + PSA(5uM)	3	A - F	p < 0.0001	***
	G	NHS + PSA(15uM)	3	A - G	p = 0.0001	***
	H	NHS + PSA(50uM)	4	A - H	p < 0.0001	***
	I	NHS + PSA(75uM)	3	A - I	p = 0.0002	***
**FigEV4E**	A	Control	3	E - A	p = 0.001	***
	B	PolySia avDP20	3	E - B	p = 0.001	***
	C	Control/HI-NHS	3	E - C	p < 0.0001	***
	D	PolySia avDP20/HI-NHS	3	E - D	p = 0.002	**
	E	Control/NHS	11			
	F	PolySia avDP20/NHS	3	E - F	p = 0.002	**
**FigEV4F**	A	NHS + PI	11			
	B	NHS + PSA(0,05uM)	3	A - B	p = 1.000	
	C	NHS + PSA(0,15uM)	3	A - C	p = 1.000	
	D	NHS + PSA(0,5uM)	3	A - D	p = 1.000	
	E	NHS + PSA(1,5uM)	3	A - E	p = 1.000	
	F	NHS + PSA(5uM)	3	A - F	p = 1.000	
	G	NHS + PSA(15uM)	3	A - G	p = 0.751	
	H	NHS + PSA(50uM)	3	A - H	p = 0.004	**
	I	NHS + PSA(75uM)	3	A - I	p = 0.008	**

Appendix figure S1

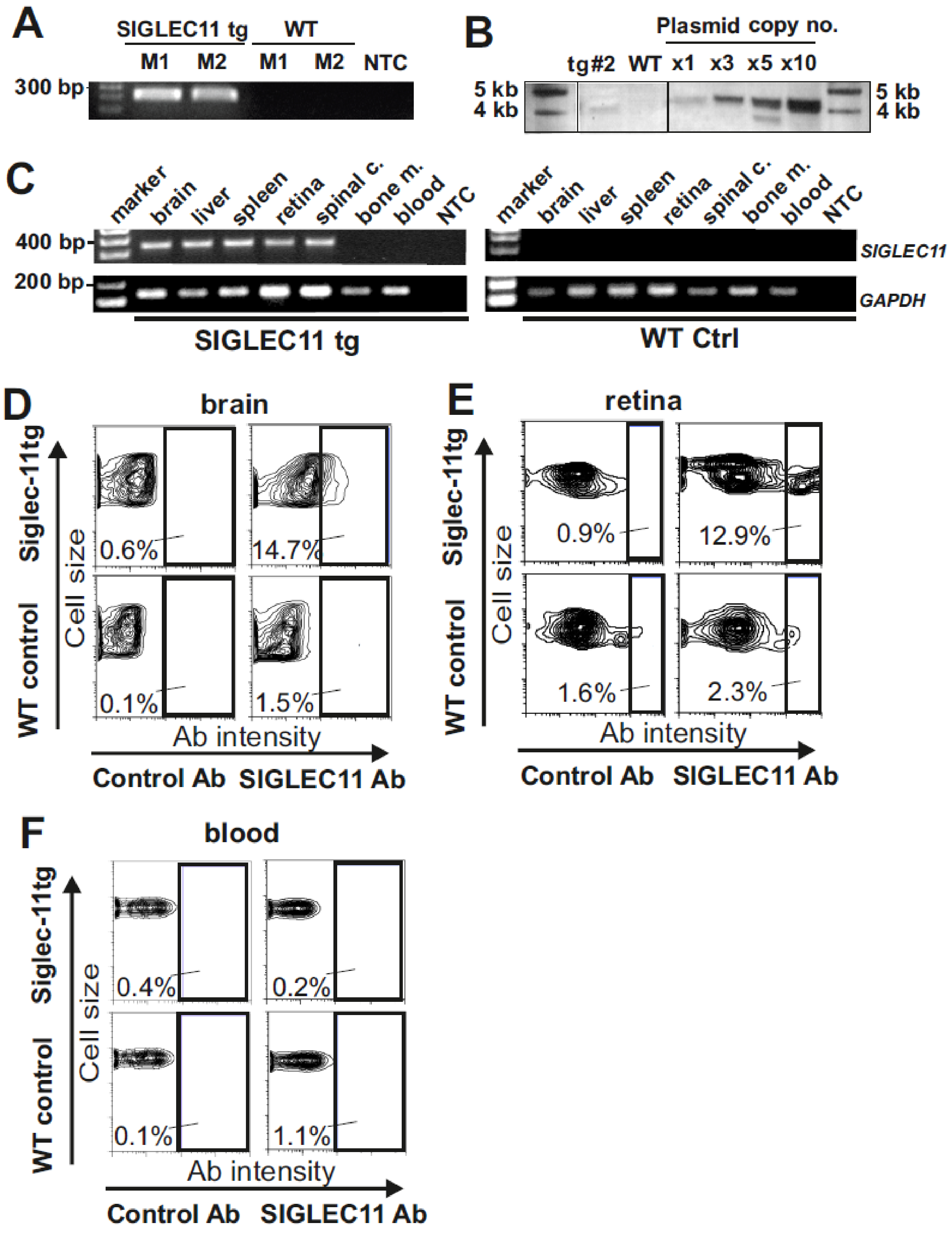

Legend to Appendix figure S1.

Generation and characterization of SIGLEC11 transgenic mice.
A Transgenic mice were generated by pro-nuclear injection of a plasmid coding the human SIGLEC11 gene driven by the mouse Iba1-promoter. Tail biopsies of SIGLEC11 transgenic (SIGLEC11 tg M1 and M2) and littermate control mice (WT M1 and M2) were analyzed by PCR with specific oligonucleotides for the targeting vector. A specific band of 252 bp for SIGLEC11 was amplified from genomic DNA of SIGLEC11 transgenic mouse lines. NTC, non-template control. Representative images out of at least three independent experiments are shown.
B Southern blot analysis of genomic mouse DNA. SIGLEC11 was detected in the biopsy of the SIGLEC11 transgenic mouse (tg #2), but not of the littermate control mouse (WT). The SIGLEC11 template plasmid DNA containing different copy numbers of the SIGLEC11 open reading frame served as a positive control.
C SIGLEC11 gene transcript analysis of different organs from SIGLEC11 transgenic (SIGLEC11 tg) and littermate control mice (WT Ctrl). Total RNA was collected and analyzed for SIGLEC11 gene transcripts. RT-PCR amplification revealed a specific band of 352 bp for SIGLEC11 from the cDNA of brain, liver, spleen, retina and spinal cord (spinal c.) from the SIGLEC11 transgenic mouse. No gene transcripts for SIGLEC11 were detected in bone marrow (bone m.) and blood and in the organs of the littermate control mouse. GAPDH served as housekeeper control. NTC, non-template control. Representative images out of at least three independent experiments are shown.
D-F Flow cytometry analysis of total tissue suspensions from brain (D), retina (E) and blood (F). SIGLEC11 expression is detected on a subpopulation of cells in the brain and retina, but not on blood cells derived from SIGLEC11 transgenic mice (SIGLEC11 tg). Littermate control mice (WT control) and an isotype control antibody (control Ab) were used as controls. Representative images out of three independent experiments are shown.

Appendix figure S2

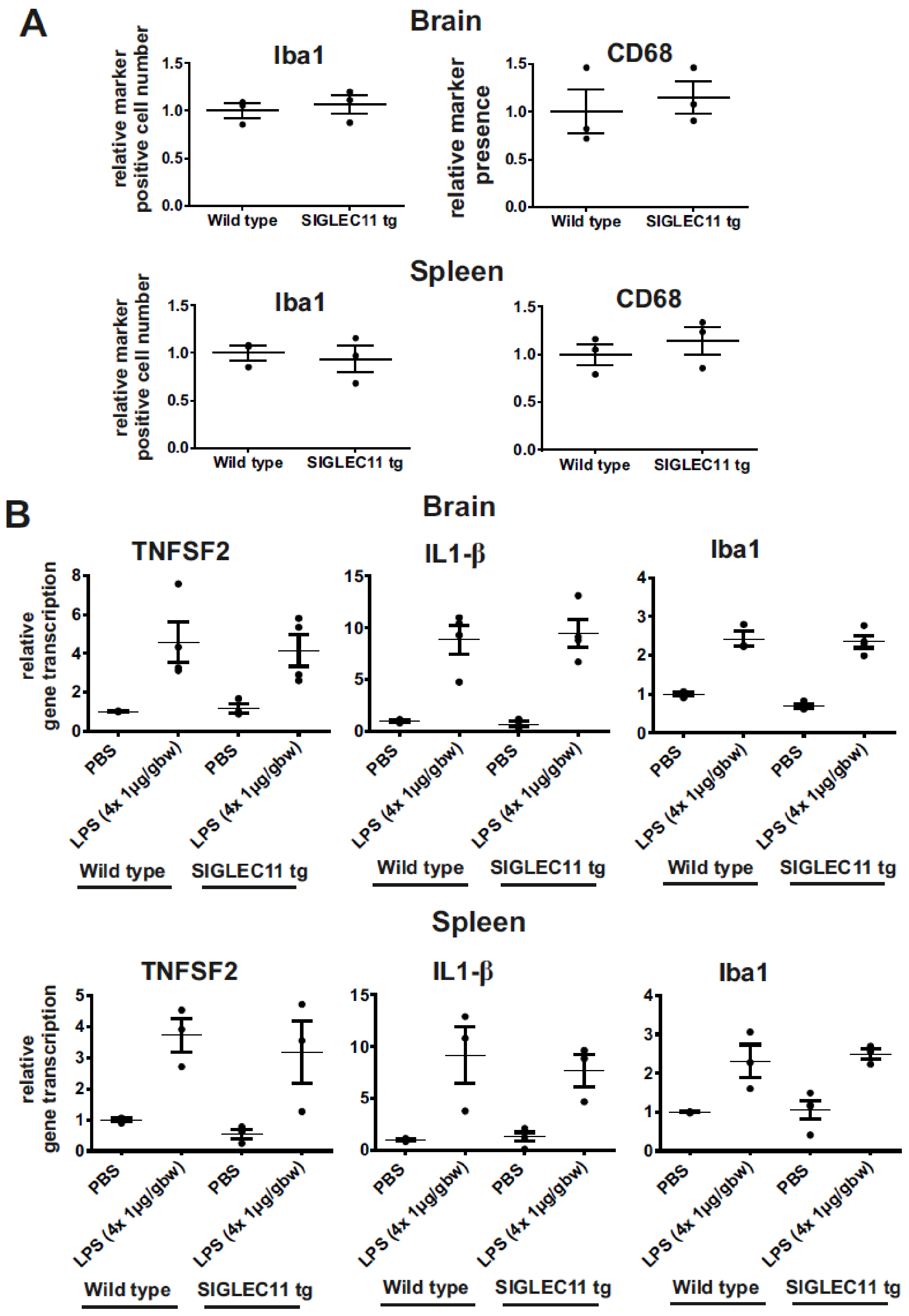

Legend to Appendix figure S2.

No change in microglia/macrophage cell number and pro-inflammatory cytokines in the brain and spleen of SIGLEC11 transgenic mice.

A Comparison of Iba1- and CD68-positive cells in brain and spleen tissue of SIGLEC11 transgenic (tg) mice and littermate controls (Wild type). No significant difference was detectable in the different organs. Data are shown as mean +/- SEM. n = 3. At least 3 pictures were taken per animal, analyzed by unpaired t-test.

B Comparison of TNFSF2, IL1-β and Iba1 transcription in LPS-injected and control SIGLEC11 transgenic (tg) and littermate mice (Wild type). Animals received four daily injections with 1 µg/gbw LPS or vehicle and were sacrificed at day five. Data show mean +/- SEM, analyzed by ANOVA followed by Bonferroni. Statistical analysis was done in relation to the wild type PBS control animals. Wild type PBS n= 3, wild type LPS n= 3, Siglec11 PBS n= 3, Siglec11 LPS n= 3.

Appendix figure S3

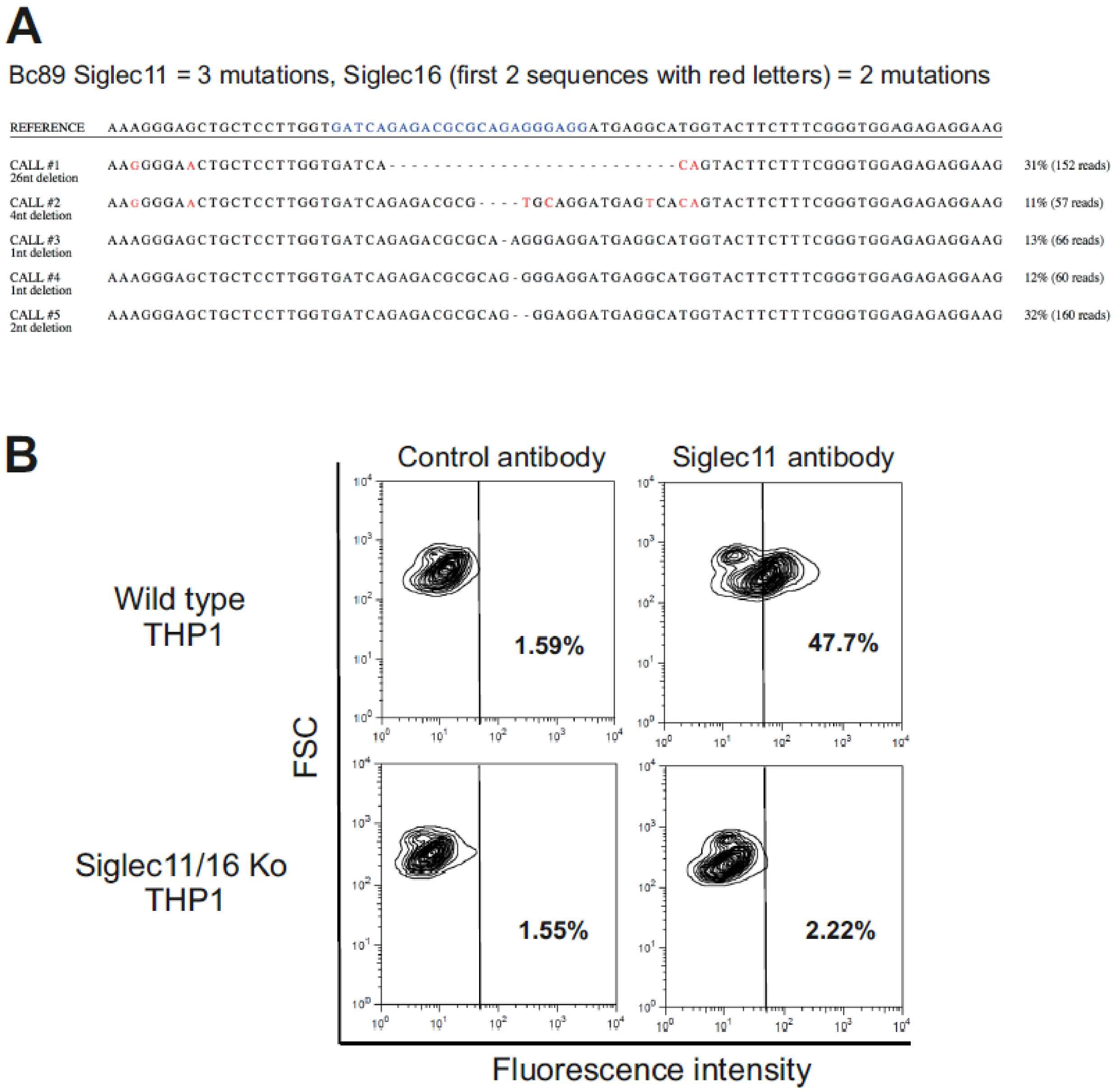

Legend to Appendix figure S3.

Validation of the Siglec11/16 knockout THP1 clone Bc89.

A Confirmation of CRISPR/Cas9-induced mutation of the SIGLEC11 and SIGLEC16 gene via sequencing. SIGLEC11 revealed 3 and SIGLEC16 2 deletions within the region of exon 1.

B Confirmation of notably reduced SIGLEC11 presence on the cell surface of THP1 clone Bc89 compared to wild type THP1 cells via flow cytometry. Representative images of at least three independent experiments are shown.

Appendix figure S4

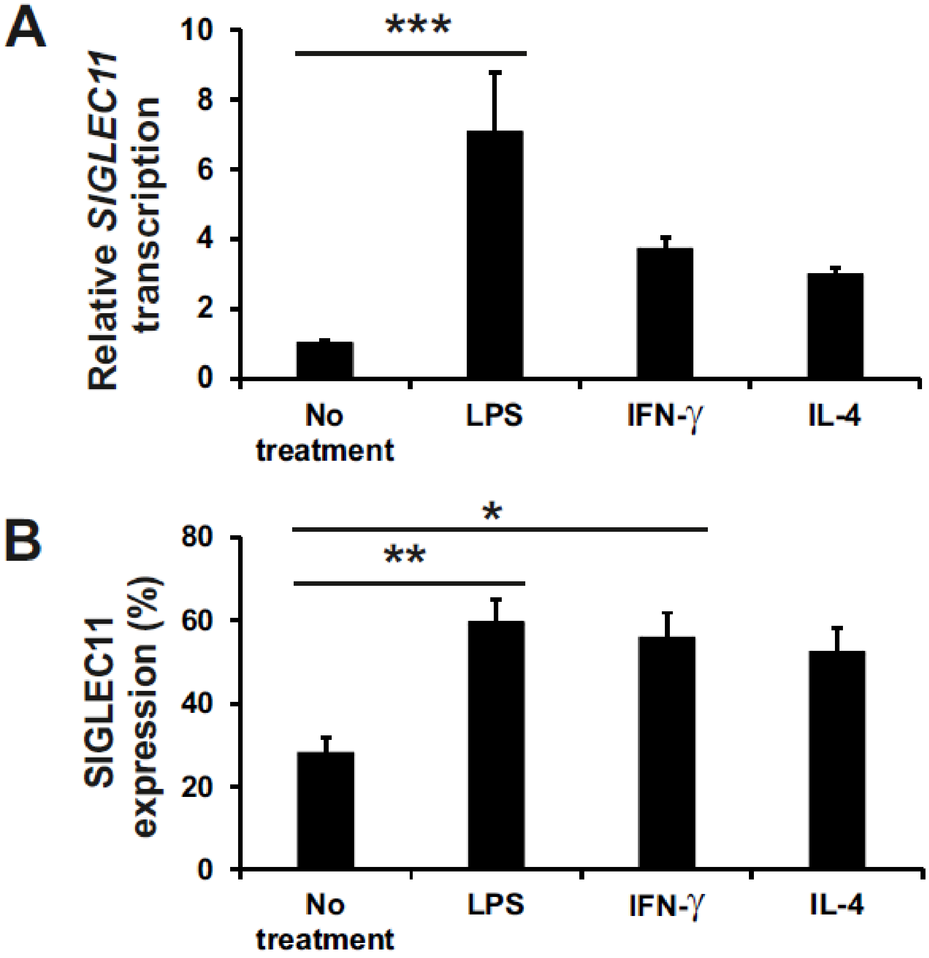

Legend to Appendix figure S4.

Inflammatory mediators increase SIGLEC11 expression in THP1-macrophages.
A Analysis of *SIGLEC11* gene transcription of THP1 macrophages stimulated for 24 hours with LPS (1 µg/ml), IFN-γ (1000 U/ml) or IL4 (20 ng/ml). LPS treatment led to a significant increase of *SIGLEC11* transcription ($p = 0.001$). Data are shown as mean +/- SEM. ANOVA followed by Bonferroni. Statistics were done with respect to the no treatment control. WT: no treatment n=7, LPS n=3 and p=0.001, IFN-γ n=3 and p=0.17, IL4 n=4 and p=0.434.
B Analysis of the regulation of SIGLEC11 expression in LPS (1 µg/ml), IFN-γ (1000 U/ml) or IL4 (20 ng/ml) -stimulated THP1 macrophages (24 hours). LPS ($p = 0.007$) and IFN-γ ($p = 0.027$) treatment led to a significant increase of SIGLEC11 cell surface presence. Data are shown as mean +/- SEM. ANOVA followed by Bonferroni. Statistics were done with respect to the no treatment control. WT: no treatment n= 4, LPS n= 4 and p=0.007, IFN-γ n=3 and p=0.027, IL4 n= 3 and p=0.059.

Appendix figure S5

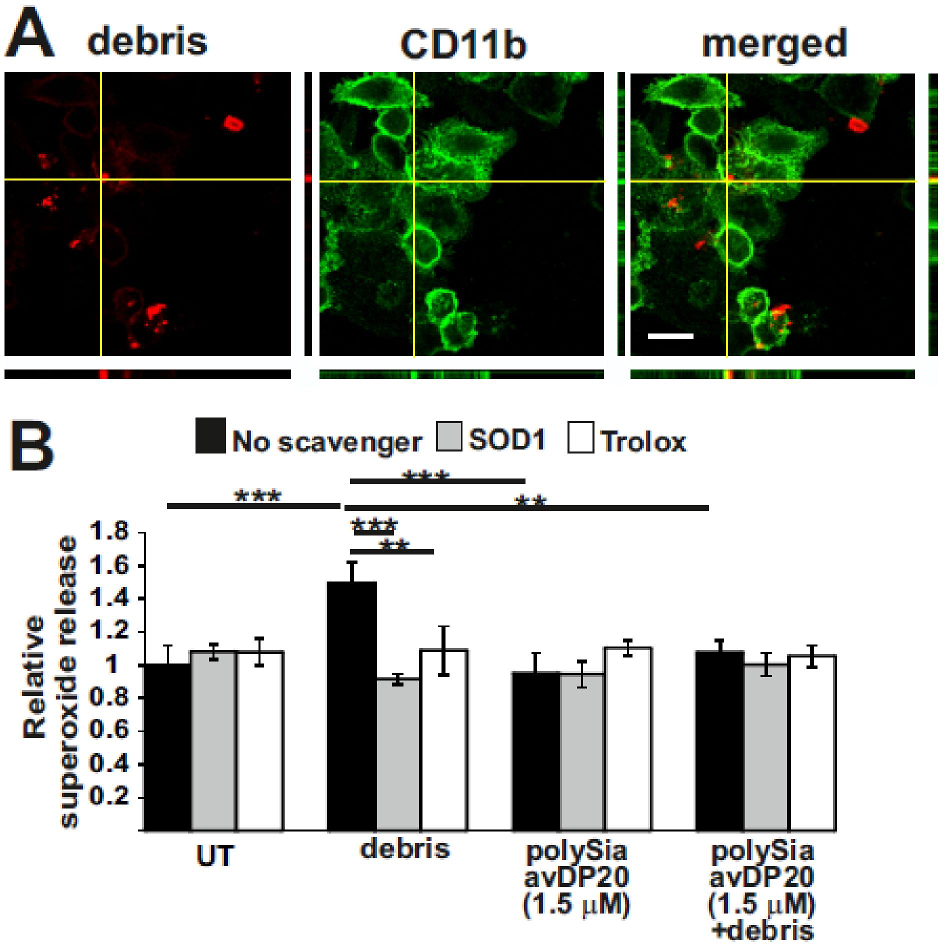

Legend to Appendix figure S5.

PolySia avDP20 inhibits superoxide production in human macrophages comparable to scavengers during phagocytosis of debris.

A Phagocytosis of drusen-like debris by human macrophages. Fluorescently labelled debris obtained from human retinal pigment epithelial cells (red) was added to the THP1-macrophage cells (green) for 1.5 hours. Cells were fixed and analyzed by confocal microscopy and 3D-reconstruction. Representative images out of at least three independent experiments are shown. Scale bar: 50 µm.

B Trolox and SOD1 scavenged the superoxide release, indicating cell surface membrane-associated production of the radicals. Data are presented as mean +/- SEM, n=5, $^{**}p \leq 0.01$, $^{***}\ p \leq 0.001$, ANOVA followed by Bonferroni. Untreated vs debris stimulated macrophages p=0.000031, debris stimulates vs polySia avDP20 stimulated macrophages p= 0.00001, debris stimulated vs debris plus polySia avDP20 stimulated macrophages p=0.002, debris stimulated vs debris plus SOD1 stimulated macrophages p=0.000004, debris stimulated vs debris plus Trolox stimulated macrophages p=0.004.
