# Supplementary material for: Polysialic acid blocks mononuclear phagocyte reactivity, inhibits complement activation, and protects from vascular damage in the retina
Source: EMBO Mol Med. 2016 Dec 22;9(2):154–66. doi: 10.15252/emmm.201606627 (PMC5286381; doi:10.15252/emmm.201606627)
Supplement: Supplementary file 2 — Expanded View Figures PDF [file EMMM-9-154-s002.pdf]

Expanded View Figures

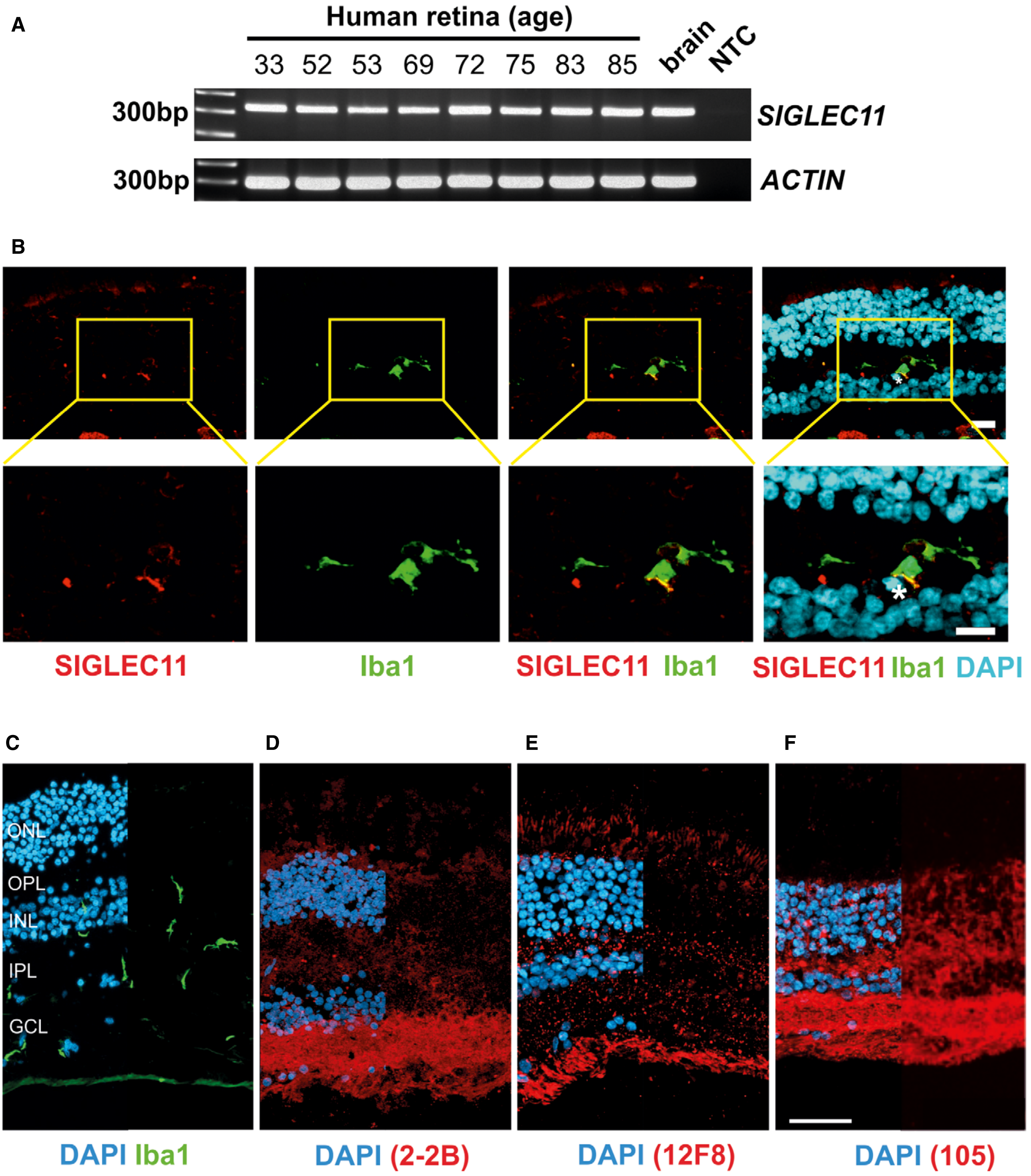

© 2016 The Authors

Figure EV1. Detection of SIGLEC11 and oligosialic/polysialic acid in human retinas.

A RT–PCR analysis of human *post-mortem* retinas from healthy donors for detection of *SIGLEC11* gene transcripts. *SIGLEC11* gene transcription at varying intensities was detected in the human *post-mortem* retina derived from eight individual donors of different age. Human brain cDNA (brain) was used as positive control. NTC, non-template control. Representative images out of at least three independent experiments are shown.

B Immunohistochemical staining of SIGLEC11 (red) and Iba1 (green) in human retina shows SIGLEC11 immunoreactivity on a subpopulation of microglial cells. Nuclei were counter-labeled with DAPI (blue). Double-labeled cell is marked by an asterisk. Representative images out of at least three independent experiments are shown. Scale bar: 10 μm.

C–F Immunostaining of human retina obtained from a healthy donor with antibodies directed against microglia (Iba1; C), polySia (D; clone 2-2B), polySia (E; clone 12F8), and oligoSia (F; clone 105). Cell nuclei were counterstained with DAPI. Iba1-positive ramified microglia were detected in the ganglion cell layer (GCL), the inner plexiform layer (IPL) of the retina, and the inner nuclear layer (C). Strong expression of all different chain lengths of oligo-/polysialic acid was detected in the GCL. Weak expression of all three chain lengths of oligo-/polysialic acid was detected in the IPL and the outer plexiform layer (OPL) of the human retina. Representative images out of at least three independent experiments are shown. Fluorescence images show merge channels of the respective antibody staining with DAPI on the left half and antibody staining alone in the right half of the image. ONL, outer nuclear layer; OPL, outer plexiform layer; INL, inner nuclear layer; IPL, inner plexiform layer; GCL, ganglion cell layer. Scale bar: 50 μm.

Figure EV2. Detection of SIGLEC11 and oligosialic/polysialic acid in the murine retina.

A RT–PCR analysis of total retinal RNA isolated from wild-type mice (wt mouse) and humanized SIGLEC11 transgenic mice (SIG11 tg mouse) for detection of *SIGLEC11* gene transcripts. Gene transcripts for *SIGLEC11* were detected in the SIG11 tg mouse, but not in wild-type (wt) control mouse. Messenger RNA derived from three different human retinas served as positive controls demonstrating inter-individual human variability. β-Actin served as a loading control. NTC, non-template control. Representative images out of at least three independent experiments are shown.

B Flow cytometry analysis of the retina from SIGLEC11 transgenic (SIGLEC11 tg) and littermate control (WT control) mice. Cells were triple-stained with antibodies directed against CD11b, CD45, and SIGLEC11. Matched isotype control antibodies were used as controls. Microglial cells and perivascular macrophages double-stained for CD11b and CD45 were gated (left graphs) and analyzed for SIGLEC11 expression levels (right graphs). SIGLEC11 expression was detected on $CD11b^+$/$CD45^+$ cells of SIGLEC11 transgenic mice, but not in littermate control mice. Representative images out of three independent experiments are shown.

C–F Immunostaining of mouse retina with antibodies directed against Iba1 (C), polySia (D; clone 2-2B), polySia (E; clone 12F8), and oligoSia (F; clone 105). Cell nuclei were counterstained with DAPI. Iba1-positive microglia were detected in the inner and outer plexiform layers of the retina. All three different species of oligoSia/polySia were detected in all retinal layers. Fluorescence images show merge channels of the respective antibody staining with DAPI on the left half and antibody staining alone in the right half of the image. ONL, outer nuclear layer; OPL, outer plexiform layer; INL, inner nuclear layer; IPL, inner plexiform layer; GCL, ganglion cell layer. Representative images of at least three independent experiments are shown. Scale bar: 50 μm.

© 2016 The Authors

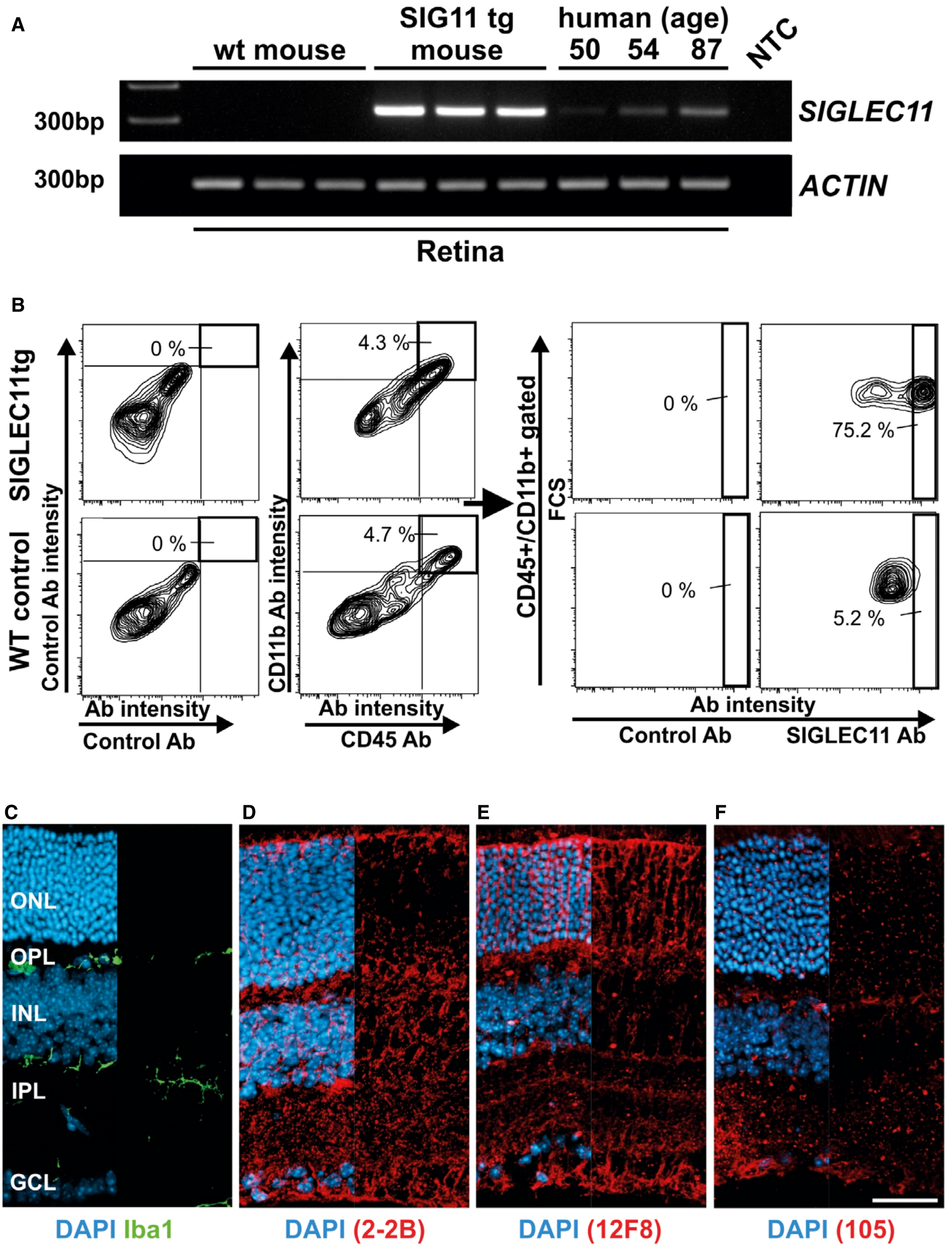

Figure EV2.

 © 2016 The Authors

Figure EV3. Higher concentration of polySia avDP20 inhibits TNFSF2 and superoxide production of mouse microglial cells.

A Analysis of relative *Tnfsf2* gene transcription and TNFSF2 protein release in a control and SiglecE knockdown murine microglial cell line. The cells were stimulated for 24 h with LPS (1 μg/ml) and polySia avDP20. Only at a concentration of 1.5 μM polySia avDP20 significantly reduced *Tnfsf2* transcription in the control microglia. Lower concentrations had no effect on the transcription level, and no significant reduction was detectable on the protein level. No response to polySia avDP20 was detectable in the knockdown cells. Data show mean ± SEM; NS = not significant, ***$P < 0.001$, ANOVA followed by Bonferroni correction. Statistical analysis was done in relation to the LPS control. WT: no treatment $n = 7$ and $P < 0.0001$, polySia avDP20 1.5 μM $n = 5$ and $P < 0.0001$, LPS $n = 7$, LPS/polySia avDP20 0.15 μM $n = 4$ and $P = 1.0$, LPS/polySia avDP20 1.5 μM $n = 4$ and $P = 0.001$. SiglecE shkd: no treatment $n = 5$ and $P < 0.0001$, polySia avDP20 1.5 μM $n = 5$ and $P < 0.0001$, LPS $n = 5$, LPS/polySia avDP20 0.15 μM $n = 4$ and $P = 1.0$, LPS/polySia avDP20 1.5 μM $n = 5$ and $P = 1.0$.

B Relative gene transcript analysis of the inhibitory effect of polySia avDP20 on *TNFSF2/Tnfsf2* was carried out via qRT–PCR. Human THP1 macrophages and murine microglia were stimulated for 24 h with LPS (1 μg/ml) and several concentrations of polySia avDP20 (0.005–15 μM). Human macrophages required around 10-fold lower concentration of polySia avDP20 to inhibit *TNFSF2* transcription than mouse microglia.

C Phagocytosis of drusen-like debris by mouse microglia. Fluorescently labeled debris obtained from RPE cells (red) was added to microglial cells (green) for 1.5 h. Cells were fixed and analyzed by confocal microscopy and 3D reconstruction. Representative images out of at least three independent experiments are shown. Scale bar: 50 μm.

D Quantification of microglial cells having ingested debris. PolySia avDP20 (1.5 μM) reduced the percentage of phagocytic cells having ingested drusen-like debris. Data are presented as mean ± SEM, $n = 4$. Debris-treated WT microglia vs. polySia avDP20 1.5 μM-treated WT microglia *$P = 0.044$, polySia avDP20 1.5 μM-treated WT microglia vs. SiglecE-kd microglia **$P = 0.009$, ANOVA followed by Bonferroni correction.

E Prevention of superoxide release in microglial cells by polySia avDP20. Cultured mouse microglia were stimulated with RPE cell debris or co-stimulated with debris and polySia avDP20. Addition of debris stimulated the production of superoxide. 1.5 μM polySia avDP20 completely prevented the release of superoxide induced by debris challenge. Data are presented as mean ± SEM, $n = 5$. Untreated WT microglia vs. debris-treated WT microglia *$P = 0.026$, polySia avDP20 1.5 μM plus debris-treated WT microglia vs. SiglecE-kd microglia *$P = 0.031$, debris-treated WT microglia vs. polySia avDP20 1.5 μM plus debris-treated WT microglia **$P = 0.004$, ANOVA followed by Bonferroni correction.

F Trolox and SOD1 scavenged the superoxide release, indicating cell surface membrane-associated production of the radicals. Data are presented as mean ± SEM, $n = 5$. Debris-treated microglia vs. debris plus SOD1-treated microglia *$P = 0.024$, debris-treated microglia vs. debris plus polySia avDP20 1.5 μM-treated microglia *$P = 0.018$, ANOVA followed by Bonferroni correction.

© 2016 The Authors

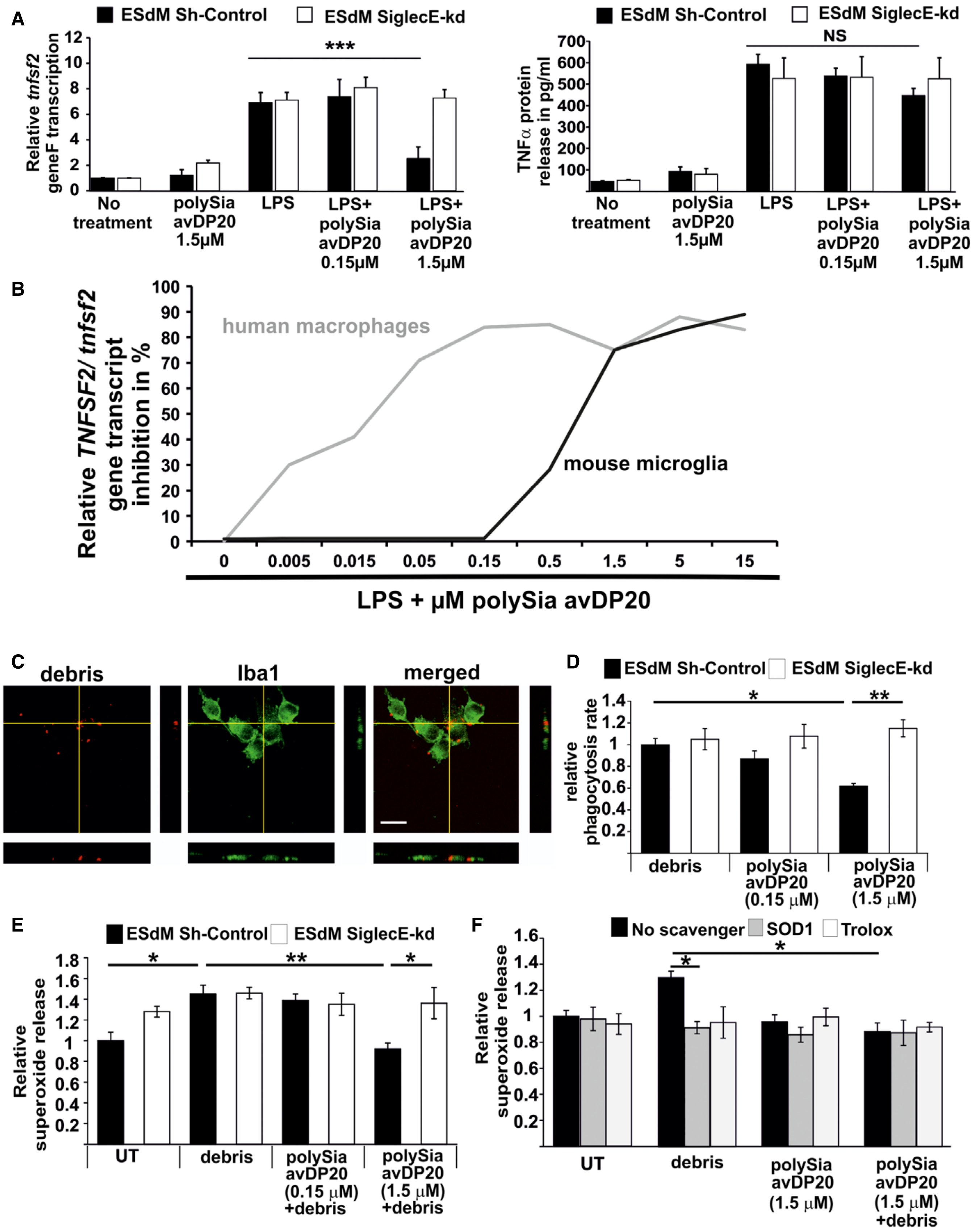

Figure EV3.

 © 2016 The Authors

Figure EV4. Interference of polySia avDP20 with complement-mediated lysis and membrane attack complex (MAC) formation.

A Flow cytometry analysis of the human complement-susceptible hepatoma cell line incubated with normal human serum (NHS), heat-inactivated normal human serum (HI-NHS), and normal human serum incubated with 1.5 μM polySia avDP20 (NHS + polySia). Cell lysis was determined by uptake of propidium iodide (PI). NHS lysed the hepatoma cell line. Lysis of the cells was inhibited by polySia avDP20. Representative images out of at least three independent experiments are shown.

B Quantification of cell lysis as determined by flow cytometry. PolySia avDP20 (50 μM) inhibited the cell lysis induced by NHS. Data show mean ± SEM. ***$P < 0.001$, ANOVA followed by Bonferroni correction. Statistical analysis was done in relation to the NHS-treated sample. Control $n = 3$ and $P < 0.0001$, polySia control $n = 8$ and $P < 0.0001$, HI-NHS $n = 8$ and $P < 0.0001$, HI-NHS/polySia $n = 6$ and $P < 0.0001$, NHS $n = 3$, NHS/polySia $n = 4$ and $P = 0.00019$.

C Quantification of cell lysis as determined by flow cytometry. PolySia avDP20 inhibited the cell lysis induced by NHS in a concentration-dependent manner. Data show mean ± SEM. ***$P < 0.001$, ANOVA followed by Bonferroni correction. Statistical analysis was done in relation to the NHS-treated sample. NHS control $n = 3$, NHS/polySia 0.05 μM $n = 3$ and $P = 1.0$, NHS/polySia 0.15 μM $n = 3$ and $P = 1.0$, NHS/polySia 0.5 μM $n = 3$ and $P = 1.0$, NHS/polySia 1.5 μM $n = 3$ and $P < 0.0001$, NHS/polySia 5 μM $n = 3$ and $P < 0.0001$, NHS/polySia 15 μM $n = 3$ and $P = 0.0001$, NHS/polySia 50 μM $n = 4$ and $P < 0.0001$, NHS/polySia 75 μM $n = 3$ and $P = 0.0002$.

D Fluorescence microscopy of membrane attack complex (MAC) formation by staining of C5b-9. MAC formation was readily visible on the hepatoma cells incubated with NHS. Incubation with HI-NHS served as a negative control. MAC formation was absent after incubation with 50 μM polySia avDP20 and NHS (NHS + polySia). Representative images out of at least three independent experiments are shown. Scale bar: 50 μm.

E Analysis of MAC formation on the surface of mouse hepatoma cells after incubation with NHS and HI-NHS with or without polySia avDP20 (50 μM). Incubation with HI-NHS served as a negative control. MAC formation was absent after incubation with 50 μM polySia avDP20 and NHS. Data show mean ± SEM. **$P < 0.01$, ANOVA followed by Bonferroni correction. Statistical analysis was done in relation to the NHS-treated sample. Control $n = 3$ and $P = 0.001$, polySia control $n = 3$ and $P = 0.001$, HI-NHS $n = 3$ and $P < 0.0001$, HI-NHS/polySia $n = 3$ and $P = 0.002$, NHS $n = 11$, NHS/polySia $n = 3$ and $P = 0.002$.

F Quantification of MAC formation on mouse hepatoma cells after incubation with NHS and different concentrations of polySia. The formation of the MAC was decreased in NHS and polySia avDP20-treated cells in a concentration-dependent manner. Data show mean ± SEM. **$P < 0.01$, ANOVA followed by Bonferroni correction. Statistical analysis was done in relation to the NHS-treated sample. NHS control $n = 11$, NHS/polySia 0.05 μM $n = 3$ and $P = 1.0$, NHS/polySia 0.15 μM $n = 3$ and $P = 1.0$, NHS/polySia 0.5 μM $n = 3$ and $P = 1.0$, NHS/polySia 1.5 μM n = 3 and $P = 1.0$, NHS/polySia 5 μM $n = 3$ and $P = 0.751$, NHS/polySia 15 μM $n = 3$ and $P = 0.004$, NHS/polySia 50 μM $n = 3$ and $P = 0.008$.

© 2016 The Authors

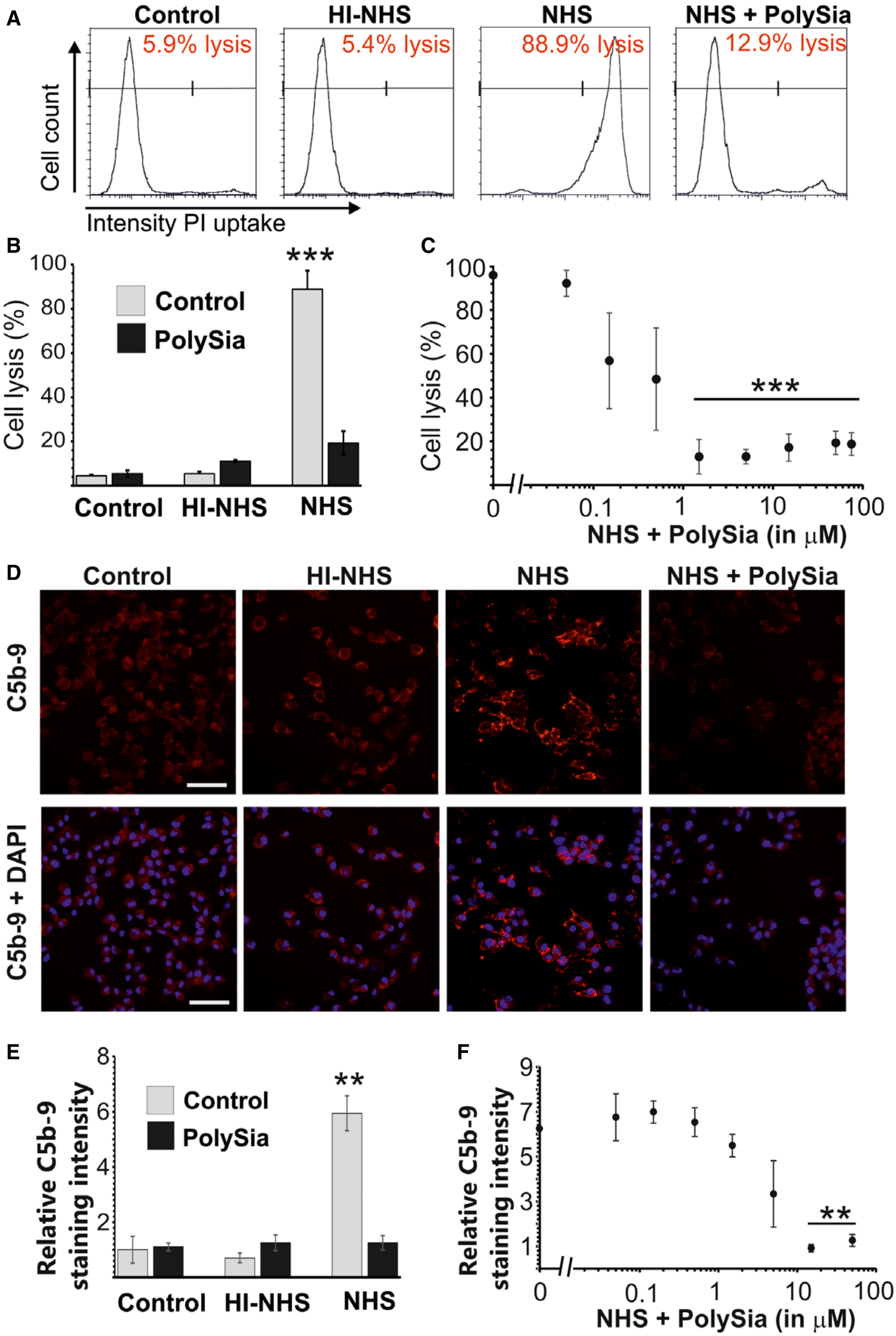

Figure EV4.

 © 2016 The Authors
